# Nursing Now and Nursing in the future: the experience of the unexpected irruptions*

**DOI:** 10.1590/1518-8345.4826.3453

**Published:** 2021-06-28

**Authors:** Dirce Stein Backes, Camila Malgarin, Alacoque Lorenzini Erdmann, Andreas Büscher

**Affiliations:** 1 Universidade Franciscana, Santa Maria, RS, Brazil.; 2 Universidade Federal de Santa Catarina, Florianópolis, SC, Brazil.; 3 Hochschule Osnabrück, Deutschen Netzwerks für Qualitätsentwicklung in der Pflege, Osnabrück, Germany.

**Keywords:** Nursing, Nursing Care, Nurse’s Role, Coronavirus, Pandemics, Nonlinear Dynamics, Enfermagem, Cuidados de Enfermagem, Papel do Profissional de Enfermagem, Coronavirus, Pandemia, Dinâmica não Linear, Enfermería, Atención de Enfermería, Rol de la Enfermera, Coronavirus, Pandemias, Dinámica no Lineales

## Abstract

**Objective:**

to carry out a theoretical reflection on the *Nursing Now Campaign* and the experience of the unexpected irruptions facing the pandemic period.

**Method:**

a theoretical-reflective study, supported by the theoretical framework of complexity thinking. It aims at understanding the dialogic between the notions of order, disorder and organization, which translate the transition from simplification to complexity of the pandemic phenomenon and its relation to the theme of *Nursing Now and Nursing in the future*.

**Results:**

the universe of phenomena is simultaneously composed of order, disorder and organization. Reasserting the central role of Nursing in the health team, facing the irruptions and uncertainties caused by the current pandemic, implies the ability to dialog with disorder and raise a new and more complex global (re)organization of the being and doing Nursing.

**Conclusion:**

in addition to answers, theoretical reflection raises new questions and irruptions. The inseparability between the notions of order and disorder in the evolutionary dynamics of the Nursing system is conceived and the promotion of even more complex levels of organization, management and Nursing assistance to achieve universal access to health is advocated.

## Abstract (pt)

**Objetivo:**

realizar reflexão teórica acerca da Campanha *Nursing Now* e a experiência das irrupções do inesperado face ao período pandêmico.

**Método:**

estudo teórico-reflexivo, apoiado no referencial teórico do pensamento da complexidade. Visa-se à compreensão da dialógica entre as noções de ordem, de desordem e de organização, as quais traduzem a passagem da simplificação à complexidade do fenômeno da pandemia e sua relação com a temática *Nursing Now* and *Nursing in the future*.

**Resultados:**

o universo dos fenômenos é tecido, simultaneamente, de ordem, de desordem e de organização. Reafirmar o papel central da Enfermagem na equipe de saúde, face às irrupções e incertezas provocadas pela pandemia em curso, implica capacidade de dialogar com a desordem e suscitar uma nova e mais complexa (re)organização global do ser e do fazer Enfermagem.

**Conclusão:**

a reflexão teórica suscita, além de respostas, novos questionamentos e novas irrupções. Concebe-se a inseparabilidade entre as noções de ordem e de desordem na dinâmica evolutiva do sistema de Enfermagem e defende-se a promoção de níveis de organização, gestão e assistência de Enfermagem ainda mais complexos para o alcance do acesso universal à saúde.

## Abstract (es)

**Objetivo:**

realizar una reflexión teórica sobre la Campaña *Nursing Now* y la experiencia de las irrupciones de lo inesperado ante el período pandémico.

**Método:**

estudio teórico-reflexivo, basado en el marco teórico del pensamiento complejo. Tiene como objetivo comprender la dialógica entre las nociones de orden, desorden y organización, que traducen la transición de la simplificación a la complejidad del fenómeno pandémico y su relación con el tema *Nursing Now* and *Nursing in the future*.

**Resultados:**

el universo de los fenómenos se entreteje, simultáneamente, de orden, desorden y organización. Reafirmar el papel central de la Enfermería en el equipo de salud, ante las irrupciones e incertidumbres provocadas por la pandemia actual, implica la capacidad de dialogar con el desorden y propiciar una nueva y más compleja (re)organización global del ser y hacer Enfermería.

**Conclusión:**

la reflexión teórica plantea, además de respuestas, nuevas interrogantes y nuevas irrupciones. Se concibe la inseparabilidad entre las nociones de orden y desorden en la dinámica evolutiva del sistema de Enfermería y se aboga por la promoción de niveles aún más complejos de organización, gestión y asistencia de Enfermería para lograr el acceso universal a la salud.

## Introduction

The *Nursing Now* global campaign, performed in collaboration with the International Nursing Council and the World Health Organization, arose to reassert the central and vital role of Nursing in the health team, especially in a year marked by the pandemic caused by COVID-19^(^ 1 ^)^. The experience of the unexpected irruptions in 2020 emphatically denotes that the Nursing professional can be the protagonist of new care technologies, booster of new health politics, and mediator of integrating and articulating processes of promotion, protection and recovery of health^(^ 2 ^)^.

The pandemic caused by the coronavirus (severe acute respiratory syndrome coronavirus 2, SARS-COV-2) quickly changed the course of daily life, tested the logic of the economy, and increased the suffering and fear of death of thousands of people^(^ 3 ^-^ 5 ^)^. Its configuration and repercussions, quintessentially complex, represent an important threat to public health and human life worldwide. No event in recent history has affected life so deeply and widely in all its dimensions as the pandemic event caused by COVID-19.

We find ourselves facing the chaos defined by the author of complex thinking, Edgar Morin, as an irreversible or volatile phenomenon, not reducible to a predictable mathematical reality. As the world becomes globalized, human minds expand more and more to follow this development, which in turn originates even more complex phenomena. In the author’s view, uncertainty, unpredictability and contradictions are part of the human condition and, in this direction, suggest solidarity and ethics as a possible way for the reconnection of the professional beings and knowledge, in this case, those of Nursing^(^ 6 ^-^ 8 ^)^.

Therefore, we are facing systemic disorder with the possibility of creating a new global organization, more visionary, prospective and integrated also in the Nursing area. The moment calls researchers and professionals in general to expand knowledge, connect, interconnect and understand the part in the whole, as well as the whole in each part and thus, find new meanings in the midst of the uncertain, the unexpected and the random. Amid disorder and chaos, new opportunities can emerge^(^ 9 ^-^ 10 ^)^. Conceiving disorder and chaos under this focus does not mean shaping the pandemic in regretting missed opportunities, but rather enhancing them so that the *Nursing Now* Campaign attains its proposed objectives.

In addition to strengthening prospective and visionary leadership, it is essential that Nursing expands the influence and dissemination of its knowledge and professional practices. It is also essential that Nursing perceives itself as connected to other areas and articulating new processes, products and services that contribute to the promotion of health and citizenship. Therefore, how to evidence and project Nursing nationally and internationally in face of the COVID-19 pandemic? The objective, based on this impulse, is to carry out a theoretical reflection on the *Nursing Now* campaign and the experience of the unexpected irruptions in the face of the ongoing pandemic period, in the light of the complexity thinking.

## Method

A theoretical-reflective study, supported by the theoretical framework of complexity thinking. It aims at understanding the dialogic between the notions of order, disorder and organization, which translate the transition from simplification to the complexity of the pandemic phenomenon and its relation to the theme of *Nursing Now and Nursing in the future*. It is conceived that complex thinking is established as a solid framework for the understanding of different social phenomena, but in particular the path of the Nursing profession now and in the future.

The author of complex thinking, Edgar Morin, invites scientists on a journey without a predefined methodological path, but encourages them to conceive complexity and develop their own strategies, based on predictable and unpredictable problems. In Morin’s conception, the method as a path is linked to the experience of the uncertain, the random and the lived by the researchers and, thus, invites them to modify their approach based on the (re)construction and the expansion of knowledge^(^ 11 ^)^. Therefore, Morin enables a methodological path in which researchers are induced to learn, to invent and to (re)create their own path, through significant interpretive processes in the here and now.

Under this focus, the present study is not based on a preconceived method, but is guided by complex thinking, which is outlined in inventing, questioning and (re)creating its own path, based on life experiences when learning, teaching and caring in Nursing. Thus, the theoretical-reflective framework consists of old and recent productions by Edgar Morin, such as books, articles or published interviews, which retain the core of complex thinking, highly evolutionary and transformative^(^ 12 ^-^ 16 ^)^. Along this path, concepts such as certainty, uncertainty, order, disorder and organization will be explored without taking them as conclusive.

## Results

The universe of phenomena is simultaneously woven of order, disorder and organization, in addition to certainties and uncertainties. Reasserting the central role of Nursing in the health team, today and in the future, facing the irruptions and uncertainties caused by the current pandemic, implies the ability to dialog with disorder and raise a new and more complex global (re)organization of the being and doing Nursing. However, this process evolves as the professionals challenge themselves to new reflections and prospective questions.

Based on the theoretical framework of complexity thinking, more specifically from Edgar Morin’s productions, two illuminating thematic categories were outlined, namely: *The inseparability between the notions of order and disorder; and Is it possible to keep predicting Nursing in the future even in the inability to understand Nursing Now?*

### The inseparability between the notions of order and disorder

In the perspective of complex thinking, the notions of order and disorder involve different levels of apprehension. While the idea of order is related to modern science, in which the sovereignty of monarchies and divine law predominated, the notion of disorder developed with the connotation of irregularity, inconstancy and instability^(^ 7 ^-^ 8 ^)^. Disorder, in other words, was associated with disturbance, deviations or any event that led to disorganization, disintegration, death.

The Nursing system developed, paradoxically, with this sovereign connotation, that order needed to prevail over disorder and, easily, any adverse advent was eliminated. This sovereign and disciplinary order, generally perceived as an absolute and irrevocable truth, was expressed in the way of caring, relating and communicating with the patient, family and health professionals in general. This same order was also visible in the hierarchical position of the professions, in which medical knowledge was superimposed as a sovereign order over the knowledge of Nursing and other health professionals.

In this paradigmatic context, disorder was reduced and diverted from any interactive possibility. Conflicts, disintegration and disorganization were generally feared, to the detriment of the disciplinary order, accepted as truth. Like disorder, death was reduced and disintegrated from the vital dynamic process. And Nursing errors are generally conducted through strict punishment and vigilance. Finally, care was fragmented and reduced to the physical and technical dimension, disintegrating it from the complex unit.

It is noted that this sovereign, disciplinary and unquestionable order had repercussions in the Nursing leadership process and in decision-making, as well as in the linear and punctual reproduction of care. In this context, everything that was predictable, orderly and controllable was valued. If this period showed important advances in Nursing, much greater they may be in the midst of the disorder caused by the pandemic, which has driven irruptions and innovations in a fraction of seconds.

### Is it possible to keep predicting Nursing in the future even in the inability to understand Nursing Now?

The Nursing Now global campaign broke out at an opportune time, when the unexpected was not yet thought about: the pandemic. We are faced with an uncertain, unpredictable and chaotic scenario, for which the political, economic, planetary and health consequences caused by social confinement cannot be predicted yet. In a recent journalistic interview, Morin states that he does not know if it is possible to expect the worst, the best or both mixed; however, he believes that we are moving towards new uncertainties^(^ 17 ^)^.

In one of his speeches, the person in charge of the World Health Organization’s Sanitary Emergencies program stated that, despite signs of hope, realism in expectations is needed. He recognizes that it is necessary to be realistic and understand that, however great the efforts of researchers to find a vaccine, it will demand a quality and safety process^(^ 18 ^)^.

In this path, it is necessary to transcend the disciplinary barriers of Nursing/health and achieve knowledge which is integrated and interconnected to different areas. This process implies dialog and negotiation with the uncertain and the random, in addition to overcoming reductionisms and, above all, the fragmented and supporting logic of Nursing care.

The pandemic moment calls us to reflect and understand that the Nursing science does not have a repertoire of absolute truths and long-lasting and unquestionable theories. The current pandemic presupposes the reform of Nursing thought and practice, based on frameworks capable of questioning, expanding and prospecting new and more complex theoretical-practical interventions, without apprehending them as truths and without taking them as conclusive.

The entire crisis brings opportunities and the possibility of starting (afresh). In a scenario of total uncertainty, curiosity and the ability to connect in search of agile and collegiate solutions will be skills increasingly required in different areas of knowledge. Therefore, it is necessary that the Nursing professional is able and willing to see the opportunities in the midst of the turbulence of adverse advents. Under the impulse of complex thinking, the pandemic teaches us to deal with adverse demands in an integrated and collaborative way and to strengthen skills such as resilience, patience, tolerance, innovation and the capacity for empathic listening.

Based on the above, some questions are presented without the intention of exhausting them in their reflection: How to train Nursing students for the unexpected, the uncertain, the unpredictable and the random? What principles and practices are necessary to sustain Nursing care as a complex unit in times of pandemic, when there is still no effective treatment and not even clarity and security in relation to tomorrow? What Nursing protocols are developed to make the random flexible and ensure the uniqueness of each human being? What new care technologies are developed to ensure the safety of patients and Nursing professionals? How are interdisciplinary connections and collaborative and supportive interdependence developed? What skills need to be encouraged in order to maintain dynamic integration and not to “sink” among the possible currents of the voyage? Ultimately, why do we need to have absolute certainties or ready recipes when we know that the route is outlined while walking?

## Discussion

The pandemic phenomenon is not treated as an invention, but rather as an event that takes place over long periods of time. Likewise, catastrophes experienced at different times are sometimes revealed by the action of fire and others by the action of water. These phenomena serve for the Earth and humanity to (re)organize themselves and for human beings to learn that the best evolutionary possibilities can arise in chaos^(^ 6 ^-^ 9 ^)^.

The different questions explained above evoke a new way of thinking, managing, caring, teaching and researching, based on complex thinking, in the sense of *complexus* - which is woven of heterogeneous and associative constituents. The universe of phenomena is inseparably the fabric of order, disorder and organization. These notions are complementary and, with regard to order and disorder, also antagonistic and even contradictory. This evolutionary dynamic denotes that complexity is a logical notion, which unites one and multiplies it into *unitas multipiex* of the *complexus* and, at the same time, complementary, antagonistic and dialogical. Under this focus, achieving the complexity of the real means conceiving mental binocularity and abandoning simplified thinking, without giving rise to new reductionisms^(^ 12 ^-^ 14 ^)^.

As it aspires to singular and multidimensional knowledge, complexity thinking is applicable to any area of knowledge. Apprehending Nursing care as a complex unit necessarily implies the expansion of the concepts of human being, life, health, environment and time - both present and future. If human beings - complex and plural, cognoscent, socio-political-cultural - have the skills to produce, evolve and transform, they also have the ability to destroy and kill, without predicting the devastating consequences^(^ 9 ^,^ 15 ^-^ 16).

Therefore, it is recognized that everything is born and ends by the action (or not) of human beings. Thus, as human beings, Nursing professionals have in their hands the driving and transforming weapon of learning, managing, teaching and caring. They also have the strength to explore new policies and contribute to universal access to health. Under this thinking, the COVID-19 pandemic, in the midst of the *Nursing Now* campaign, came to reiterate that this biological war of the pandemic is not fought with nuclear weapons or firearms, but with care in its unique and multidimensional aspect.

Far beyond learning new content, validations and theoretical reproductions, it is necessary for Nursing professionals to learn in the midst of everyday order and disorder, the true meaning of citizenship, cooperation and solidarity. To (re)learn their own path amid mistakes, successes and random situations. To stimulate imagination and creativity in the search for new opportunities and possibilities and to assume their social role and be the protagonists of their own stories.

The sick, exhausted and disintegrated society no longer supports the same dynamics and the social model that reproduces knowledge and practices. The current moment demands renewed skills, attitudes and frameworks from each citizen and professional. It is opportune to learn the lessons resulting from the pandemic, which will be overcome, unfortunately with the sacrifice of many lives, including the expressive number of Nursing professionals. It is necessary to recognize that humanity needs to take a new course. Supportive solidarity is not enough, which is summarized in specific actions, but solidarity networks are needed, with a transforming character, educational strength, and a path to integral development^(^ 15 ^-^ 16 ^)^.

The *Nursing Now* campaign emerged at this historic moment to increase investment in improving education, professional development, regulation, and working conditions for nurses. To increase the influence on the national and international policies and the number of nurses in leadership positions with more opportunities for professional and social development. To increase the evidence to support policies and improve the dissemination of effective and innovative Nursing practices. To tell the world that Nursing is an essential profession and the driver of the health systems, both today and in the future^(^ 19 ^-^ 20 ^)^.

The contributions of this study to the advancement of scientific knowledge in the theme in question are related to the perception that Nursing is a dynamic, agile and flexible profession to the (re)invention and (re)construction of knowledge and practices, even if amid the uncertain, the unexpected and the random. The experience of the outbreaks of the unexpected in the face of the pandemic period demonstrates that Nursing needs to be able and willing to see opportunities in the midst of the turbulences of adverse adventures, in addition to being able to not separate what is inseparable and reduce to a single element what is one and multiple, such as Nursing/health care. In short, it is shown that Nursing is capable of contributing to social development, which has to do with the expansion of spaces and real opportunities for individuals, families and communities.

*Nursing in the future*: what to expect and look for in times of a pandemic? A vaccine or a proven therapy for its prevention and treatment? The (re)significance of human and social behaviors and attitudes? Reconciliation with nature that provides the essential elements for humanity’s survival? An explosion of creativity, technological reinvention, communication between the distant and taking advantage of new opportunities? A new thinking that can lead humanity to a new social dynamic, in line with its achievements and advances? A new sociological knowledge to convince humanity that life is not a simple mathematical product? A renewed and non-negotiable solidarity to face individualism? Reflexive self-criticism in which the real values and principles of the Nursing profession are (re)learned? A Nurse leader, entrepreneur and protagonist of their own story?

As a limitation of this study, the proposals of questions in the light of the complexity thinking is considered, without the possibility of answering them with specific responses and linear inflections. Thus, it is hoped that the questions will give rise to new reflections and enable a new global organization, more visionary, prospective and systemic.

## Conclusion

The theoretical reflection about the Nursing Now campaign in the face of the pandemic caused by COVID-19, in addition to timely and linear responses, raises new questions and new irruptions. *Nursing Now* and *Nursing in the Future* demonstrates that it is necessary to invest in expanding the role of Nurses as professionals in advanced practice, in prospective leadership, and expecting the unexpected without apprehending and understanding the present moment. In addition to certainties and absolute truths, it is important that Nursing stimulates imagination and creativity in the search for new alternatives, spaces and dialogs with the different professional knowledge areas.

In short, the inseparability between the notions of order and disorder in the evolutionary dynamics of the Nursing system is conceived. And the promotion of even more complex levels of organization, management and Nursing care to achieve universal access to health is advocated.
